# 2317 Uterine serous carcinoma is associated with a high risk of venous thromboembolism regardless of latency from surgical staging

**DOI:** 10.1017/cts.2018.193

**Published:** 2018-11-21

**Authors:** Gregory M. Gressel, Lauren Turker, Shayan Dioun, Nicole S. Nevadunsky

**Affiliations:** 1 Albert Einstein College of Medicine

## Abstract

OBJECTIVES/SPECIFIC AIMS: Patients with gynecologic cancer are known to have an increased risk of venous thromboembolism (VTE) in the postoperative period secondary to hypercoagulability from both malignancy and pelvic surgery. Recent literature suggests that chemotherapy itself may be thrombogenic and prophylaxis may be beneficial in ambulatory patients receiving chemotherapy. Although extended VTE prophylaxis is commonly given after surgical staging, administration of prophylactic anticoagulation during chemotherapy or radiation treatment is not routinely performed. This study seeks to characterize risk factors and timing of VTE in a cohort of women diagnosed with uterine serous carcinoma (USC). METHODS/STUDY POPULATION: After institutional review board approval, a cross-sectional study was performed of all women diagnosed with USC between January 1999 and January 2016 at Albert Einstein College of Medicine. Data analysis was performed using Stata version 14.2 (Stata Statistical Software: Release 14, 2015. College Station, TX: StataCorp LP). Baseline clinical data was analyzed to calculate descriptive statistics. Normality of continuous variables was visually assessed and if no substantial violations were noted, data was reported as means±standard deviations. Otherwise, they were reported as medians with interquartile ranges. Categorical data was presented as number of patients with percentages. Bivariate analysis was performed to assess the association between clinical variables and diagnosis of VTE. Continuous variables (age, body mass index, number of risk factors for VTE) were visually assessed for normality. Levene’s test was used to assess for equal variance among groups. If no substantial violations were noted, means and standard deviations were calculated using 2 sample *t*-test for equal variance. Variables violating normality assumptions were analyzed using the Mann-Whitney *U*-test, calculating medians and interquartile ranges. Categorical and dichotomous variables (VTE risk factors, race, stage) were examined using the χ^2^ test or Fisher’s exact test (if expected values for more than 20% of cells were less than 5). Odds ratios were reported with 95% confidence intervals. Using a backwards stepwise elimination approach, a multivariable logistic regression model was fit to accurately examine association of risk factors with VTE, adjusting for other covariates. The resulting model was assessed for calibration and discrimination using Hosmer-Lemeshow test for goodness of fit, classification table, and ROC curve. Regression diagnostics were run in order to identify potentially influential covariate patterns in the model. First-order interactions were assessed for using product interaction terms (interaction defined as *p*-value for the likelihood ratio test <0.05). The resulting model was assessed for calibration and discrimination using Hosmer-Lemeshow test for goodness of fit, classification table, and ROC curve. A Cox proportional hazards model was also fit in order to examine the association between individual covariates and time to clot development. Log-rank testing was performed to compare survivorship experience by groups and survivorship curves were generated using the Kaplan-Meier method. Assumptions of the proportional hazards model was confirmed visually using log-log plots and goodness of fit assessment. RESULTS/ANTICIPATED RESULTS: A total of 413 patients were identified for inclusion in the study. The majority of patients (83%) were of non-White race. Bivariate analysis revealed no significant associations between age, BMI, or race with diagnosis of VTE (*p*=0.75, 0.49, and 0.28, respectively). Patients who had more than 2 risk factors for VTE had a significantly increased likelihood of VTE diagnosis (*p*=0.02). There was a highly significant association between stage of USC and diagnosis of VTE (*p*=0.005). Patients with stage III and stage IV cancer were 2.4 and 3.5 times more likely to develop VTE than patients with stage I cancer (95% CI: 1.09–5.30, 1.74–6.83, respectively). Of the 70 patients who were diagnosed with VTE, most were not postoperative (64.3%) and a large proportion developed clots while receiving chemotherapy (35.7%). Patients who developed VTE while on chemotherapy had a median Khorana score of 1 (IQR: 1, 2). In logistic regression modeling examining association of VTE with potential risk factors, covariates selected as significant for inclusion at the *p*<0.25 level included cancer stage, composite number of risk factors, diabetes, hypertension, cardiovascular disease (CVD), and COPD. Composite risk score was identified to be a potential confounder of the relationship between individual risk factors and development of clot and was therefore left in the model for adjustment. After adjusting for other covariates, only stage 4 disease (OR: 2.66, 95% CI: 1.53, −4.64) and hypertension (OR: 2.90, 95% CI: 1.14–7.36) were associated with development of VTE and were included in the final model. No concerning violation of assumptions of logistic regression or interaction was identified. The Hosmer-Lemeshow goodness of fit test identified that the model was well-fit using 10 groupings (*p*=0.35) and receiver operator characteristic testing showed that the model had acceptable discrimination with a ROC value of 0.7. The final model was found to classify 83.1% of participants correctly. Regression diagnostics identified 4 potentially influential covariate patterns. These patterns were eliminated from the model and no meaningful differences were noted. Patients contributed a total of 16,414 person months of analysis time in study follow-up. A negative, linear association was noted between stage of cancer and time to clot development. Long-rank testing revealed a significant difference in failure by stage of disease (*p*<0.001) and presence of hypertension (*p*=0.03). Cox proportional hazard modeling revealed that after adjustment for other covariates, only cancer stage and the presence of cardiovascular disease were significantly associated with time to failure. Patients with cardiovascular disease had a 2.02-fold increased risk of CVD compared to those without CVD (95% CI: 1.16–3.47). Those with stage 3 and 4 cancer were 3.19 (95% CI: 1.53-6.64) and 8.05 (95% CI: 4.11–15.78) fold more likely to develop VTE compared to those with stage 1 disease, respectively. DISCUSSION/SIGNIFICANCE OF IMPACT: Our study demonstrated that patients with USC are at high risk of developing VTE at all time points after their disease diagnosis, not just those who have undergone recent surgery. This risk is highest for women with hypertension, CVD, and stages III and IV disease. The fact that patients who developed clots on chemotherapy had an average Khorana score of 1, suggesting that they would not have been successfully risk stratified using previously published tools. To the best of our knowledge, this is the first study to report a high hazard for VTE in patients with serious endometrial cancer even several months after surgical staging. Although this is a retrospective study and cannot make inferences about VTE incidence, it generates the hypothesis that extended VTE prophylaxis may be beneficial in this cohort of patients regardless of their latency from surgical staging. Large randomized studies are needed to test this hypothesis.

